# Estimating frontal and parietal involvement in cognitive estimation: a study of focal neurodegenerative diseases

**DOI:** 10.3389/fnhum.2015.00317

**Published:** 2015-06-04

**Authors:** Teagan A. Bisbing, Christopher A. Olm, Corey T. McMillan, Katya Rascovsky, Laura Baehr, Kylie Ternes, David J. Irwin, Robin Clark, Murray Grossman

**Affiliations:** ^1^Penn Frontotemporal Degeneration Center, Department of Neurology, University of Pennsylvania, Philadelphia, PAUSA; ^2^Department of Linguistics, University of Pennsylvania, Philadelphia, PAUSA

**Keywords:** cognitive estimation, behavioral variant frontotemporal degeneration, corticobasal syndrome, posterior cortical atrophy, prefrontal cortices, parietal cortices

## Abstract

We often estimate an unknown value based on available relevant information, a process known as cognitive estimation. In this study, we assess the cognitive and neuroanatomic basis for quantitative estimation by examining deficits in patients with focal neurodegenerative disease in frontal and parietal cortex. Executive function and number knowledge are key components in cognitive estimation. Prefrontal cortex has been implicated in multilevel reasoning and planning processes, and parietal cortex has been associated with number knowledge required for such estimations. We administered the Biber cognitive estimation test (BCET) to assess cognitive estimation in 22 patients with prefrontal disease due to behavioral variant frontotemporal dementia (bvFTD), to 17 patients with parietal disease due to corticobasal syndrome (CBS) or posterior cortical atrophy (PCA) and 11 patients with mild cognitive impairment (MCI). Both bvFTD and CBS/PCA patients had significantly more difficulty with cognitive estimation than controls. MCI were not impaired on BCET relative to controls. Regression analyses related BCET performance to gray matter atrophy in right lateral prefrontal and orbital frontal cortices in bvFTD, and to atrophy in right inferior parietal cortex, right insula, and fusiform cortices in CBS/PCA. These results are consistent with the hypothesis that a frontal-parietal network plays a crucial role in cognitive estimation.

## Introduction

Throughout the course of a day, individuals make decisions on the basis of estimations — Do I have enough gas to get to work this morning? How long will my afternoon meeting take? How much bread do I need for the week? Our decisions regarding what we buy, how we plan our day, and any of a number of other activities are strongly influenced by quantities that we calculate using estimates based on relevant knowledge. In this study, we assess the cognitive and neuroanatomic basis for deficits in quantitative estimation in groups of patients with focal neurodegenerative disease in frontal and parietal cortex.

Cognitive estimation is the strategic process of generating a mental approximation based on available but incomplete information (Shallice and Evans, 1978). Beyond the necessary semantic knowledge of the relevant concepts and working memory needed to maintain relevant information in an active state, cognitive estimation requires quantitative reasoning about familiar concepts and probabilistic processes in the face of imprecise information in order to develop a reasonable final estimated quantity (Shallice and Evans, 1978; Bullard et al., 2004). There appear to be two major components to cognitive estimation: executive resources and number knowledge. These components facilitate the integration of information from semantic memory using strategic reasoning skills to derive a probabilistic evaluation, a process that is central to cognitive estimation. For example, when estimating how long it will take to read this article, one must identify and retrieve the appropriate knowledge about reading from semantic memory, and integrate this with quantitative information about reading speed, to derive an appropriate estimate (e.g., it’s an academic article with small font so it might take longer than most reading).

In the current study, we sought to identify the neuroanatomic areas contributing to the neural network supporting cognitive estimation. Based on functional imaging studies in healthy adults and patients with focal brain damage, we hypothesize that these two components depend in part on two interacting brain regions.

Dorsolateral prefrontal cortex (dlPFC) is implicated in executive functioning (Duncan and Owen, 2000; Aron et al., 2004). Seem to be of particular importance for cognitive estimation. fMRI studies of healthy adults engaged in tasks requiring probability show activation in dlPFC (Casey et al., 2001). An fMRI study examining the neural correlates of tactile estimation showed greater levels of activation in dlPFC during a texture estimation task as compared to a nearly identical task not requiring any estimation (Kitada et al., 2005). Complementary to the neuroimaging evidence, individuals with frontal lobe damage demonstrate severe limitations in cognitive estimation abilities (Shallice and Evans, 1978). Smith and Milner (1984) showed impairment following right-lateralized dlPFC damage in patients with surgical treatment for epilepsy.

To examine the role of dlPFC in cognitive estimation, we assessed estimation abilities in non-aphasic individuals with the behavioral variant frontotemporal dementia (bvFTD), a neurodegenerative condition that is associated with GM atrophy encompassing dlPFC (Rosen et al., 2002; Grossman et al., 2004) and frontal WM disease (Whitwell et al., 2010a). Symptomatically, bvFTD is characterized by executive impairment as well as behavioral disinhibition and personality changes (Kramer et al., 2003; Libon et al., 2006; Rascovsky et al., 2011; Possin et al., 2013). We assessed cognitive estimation in these patients to minimize confounds associated with semantic knowledge, visuospatial and language abilities where their performance is generally preserved, but show limitations in reasoning, organization, and social judgment. Given the collective evidence of estimation deficits in people with executive dysfunction, and the association of these deficits with dlPFC damage, we expected bvFTD patients to be impaired compared to healthy controls in a test of cognitive estimation, and that their impaired cognitive estimation performance would relate to cortical atrophy including at least right dlPFC.

Number knowledge is also necessary to produce appropriate quantitative estimations. This is the domain of knowledge over which cognitive estimations often operate. Numerical knowledge is said to involve an analog number system that depends in part on the representation of ratios between quantities on a logarithm-like number line (Dehaene, 1997). Some have argued that the representation of precise numbers larger than 4 may depend in part on an external algorithm involving language (Dehaene et al., 1999), although we have shown that number knowledge is compromised in non-aphasic patients with CBS and PCA (Koss et al., 2010; Spotorno et al., 2014). Multiple fMRI studies using a variety of techniques have demonstrated that the parietal lobe, and particularly the intraparietal sulcus and adjacent inferior parietal lobule, play a crucial role in the representation of number knowledge (Piazza and Dehaene, 2004; Pinel et al., 2004; Danker and Anderson, 2007; Nieder and Dehaene, 2009). This includes knowledge of quantity that is mediated both symbolically by Arabic numerals and non-symbolic representations of number such as quantities of filled circles. Furthermore, the inferior parietal lobule seems to be associate with magnitude and quantitative processing, possible because of the spatial magnitude component involved in these processes. An fMRI study tested healthy volunteers during number comparisons and showed bilateral activation of the inferior parietal lobes, with higher activation on the right side (Chochon et al., 1999). Dehaene et al. (1999) also found increased fMRI and ERP activation in the inferior parietal lobe during approximation calculations as compared to exact calculations, which they attributed to non-linguistic numerical processing during approximations.

In the present study, we assessed the quantitative or numeric component of cognitive estimation in patients with parietal disease, including CBS and PCA. CBS is an extrapyramidal disorder with involuntary movements associated with basal ganglia disease and a variety of clinical features attributable to parietal disease including apraxia and cortical sensory loss (Murray et al., 2007; Armstrong et al., 2013). PCA is a variant of Alzheimer’s disease with visuospatial deficits due to parietal–occipital disease (Crutch et al., 2012). We have shown that patients with CBS and PCA have significant deficits with number knowledge, including impairments on measures involving single-digit calculations and Arabic numeral-dot matching (Koss et al., 2010; Morgan et al., 2011). Numerical and quantitative processing, crucial components of cognitive estimation, are associated with parietal lobe functioning, therefore, we expected that CBS and PCA patients with parietal damage would also show deficits in cognitive estimation.

To evaluate the specificity of the hypothesized frontal and parietal contributions to cognitive estimation we additionally evaluated patients with mild cognitive impairment (MCI). MCI is characterized by mild memory impairments (Feldman and Jacova, 2005; Albert et al., 2011) and is typically considered a prodromal form of Alzheimer’s disease (Boyle et al., 2006; Gauthier et al., 2006; Meyer et al., 2007). Critically, MCI patients have relatively preserved executive function and intact number knowledge (Zamarian et al., 2007; Aretouli and Brandt, 2010); therefore, despite cognitive impairments and likely neurodegenerative disease, we hypothesize that these patients will have relatively preserved cognitive estimation.

Complex behaviors such as cognitive estimation depend on multiple GM nodes that are integrated by WM projections. dlPFC and intraparietal sulcus hypothesized to play a role in cognitive estimation may be linked by dorsal and ventral WM streams. The dorsal stream is mediated by the superior longitudinal fasciculus (SLF), and the ventral stream by the inferior frontal-occipital fasciculus (IFO) or some combination of the uncinate fasciculus (UNC) and inferior longitudinal fasciculus (ILF). To help define the WM projections integrating the neuroanatomic network underlying cognitive estimation, we also obtained diffusion-weighted imaging studies. We expected that some combination of dorsal and ventral stream projections would also be implicated in cognitive estimation by imaging studies.

## Materials and Methods

### Participants

Thirty-nine patients with a targeted neurodegenerative disease, including patients with bvFTD (*N* = 22) or CBS/PCA (*N* = 17), 11 brain-damaged controls with MCI not involving the frontal lobe or the parietal lobe, and 25 age- and education-matched healthy controls, participated in the experiment. All participants were native English speakers. Patients were diagnosed by board-certified neurologists (M.G. and D.J.I.). bvFTD diagnosis was verified through a consensus procedure using published criteria (Rascovsky et al., 2011). The CBS/PCA group consisted of 11 patients diagnosed with CBS and six PCA patients. Consensus verification of CBS used published criteria (Armstrong et al., 2013), while verification of PCA was based on reports from the literature (e.g., Crutch et al., 2012, 2013a,b). As bvFTD patients can develop language impairments associated with semantic variant primary progressive aphasia (svPPA), any patients with symptomatic evidence of svPPA were excluded from the sample population. A brain-damaged control group consisted of patients with MCI and were diagnosed using published criteria (Albert et al., 2011). Other types of dementia such as vascular disease, head trauma or hydrocephalus were excluded through clinical evaluation. Patients with primary psychiatric or medical diagnoses that can impact cognition were excluded. Some patients may have been taking small doses of medically necessary medications such as non-sedating anti-depressants. Control subjects confirmed their status through negative self-report of a neurological and psychiatric history. Study participation was voluntary and in accordance with the informed consent procedures approved by the University of Pennsylvania Institutional Review Board.

Participants’ overall cognitive status was assessed using the MMSE (Folstein et al., 1975). Only patients with an MMSE score of 15 (out of 30 possible points) or above were included in the research sample, in order to restrict the patient participants to mild or moderate levels of dementia. Controls were required to score at least 28 on the MMSE to participate. Demographics for each group are summarized in **Table 1**. All three dementia groups had lower mean MMSE scores than the control group [χ ^2^(3) = 34.28, *p* < 0.001]. Comparison of the three dementia groups revealed that these groups did not differ in their MMSE scores [χ ^2^(2) = 4.20, *p* = 0.12]. To develop a neuropsychological profile for our focal patient groups (bvFTD and CBS/PCA), we assessed cognitive abilities in the domains of executive, visuospatial, language and memory functioning using the PBAC (Libon et al., 2011; Avants et al., 2014). Moreover, to assess comprehension of the verbal stimulus materials, we also administered the BNT (Kaplan et al., 1983) and the PPT (Howard and Patterson, 1992) to these patient groups. The neuropsychological characteristics of these groups are summarized in **Table 2**. The patient groups also did not differ in their scores on the PBAC scales [Executive Scale: *U*(29) = 96.00, *p* = 0.61; Visual Scale: *U*(28) = 63.50, *p* = 0.08; Language Scale: *U*(26) = 77.50, *p* = 0.56], with the exception of the behavioral scale for which bvFTD patients showed more severe behavioral ratings than CBS/PCA patients [*U*(28) = 0.00, *p* < 0.001].

**Table 1:** Mean (SD) of group demographic characteristics.

	bvFTD	CBS/PCA	MCI	Healthy seniors
*N* (male/female)	20/2	9/8	7/4	11/14
Age (years)	64.77 (7.71)	64.41 (7.04)	64.55 (10.72)	67.96 (8.34)
Education (years)	16.36 (3.02)	15.24 (2.11)	15.82 (3.16)	15.76 (1.99)
MMSE (maximum = 30)	25.68 (2.93)	23.06 (5.06)	22.91 (4.13)	29.04 (0.69)

*bvFTD, behavioral variant frontotemporal dementia; CBS, corticobasal syndrome; PCA, posterior cortical atrophy; MCI, mild cognitive impairment; MMSE, mini-mental state examination (Folstein et al., 1975).*

**Table 2:** Mean (SD) of patient neuropsychological characteristics.

	bvFTD	CBS/PCA
PBAC Executive Scale (maximum = 17)	7.94 (3.51) (*n* = 20)	6.78 (3.31) (*n* = 11)
PBAC Behavioral Scale (maximum = 18)	8.75 (2.99) (*n* = 20)	17.6 (0.52) (*n* = 10)
PBAC Visual Scale (maximum = 18)	15.63 (2.11) (*n* = 19)	11.09 (6.63) (*n* = 11)
PBAC Language Scale (maximum = 19)	16.28 (2.79) (*n* = 18)	15.1 (4.43) (*n* = 10)
BNT (maximum = 30)	25.36 (3.82) (*n* = 22)	23.75 (5.94) (*n* = 16)
PPT Words (maximum = 26)	24.00 (1.79) (*n* = 11)	22.77 (2.20) (*n* = 13)
PPT Pictures (maximum = 26)	24.36 (2.20) (*n* = 11)	24.17 (2.29) (n = 12)

*PBAC, Philadelphia Brief Assessment of Cognition (Libon et al., 2011; Avants et al., 2014); BNT, Boston naming test (Kaplan et al., 1983); PPT, pyramids and palm trees (Howard and Patterson, 1992); bvFTD, behavioral variant frontotemporal dementia; CBS, corticobasal syndrome; PCA, posterior cortical atrophy.*

### Behavioral Materials and Procedures

The BCET, originally developed by Bullard et al. (2004) was verbally administered to each participant as one measure in a larger battery that tested decision-making abilities and executive functioning.

The BCET asks participants to mentally calculate numerical approximations of imprecise quantitative values based on relevant information (e.g., How many slices of bread are there in a one pound loaf?). The 20-item task includes estimations across four modalities: quantity, weight, distance, and time. The task instructions emphasize that participants often will not be able to give an exact answer, but should instead provide their “best guess.” They are asked to answer with a specific number, not a range; and, they are asked to specify the units of their answer (e.g., 30 slices of bread). Whenever participants answered with a range or without specifying a unit of measurement, they were prompted to be more specific.

Participants’ responses were transformed to *Z*-scores based on published normative data (Bullard et al., 2004). As there were no a priori predictions with regard to the direction of estimation errors (i.e., over versus under estimation), the absolute values of the calculated Z-scores were used for analysis. The means of each participant’s *Z*-scores for all BCET responses were calculated and analyzed. Three patients were identified as outliers based on a mean *Z*-score greater than 5.0. These patients were excluded from all analyses reported herein. All mean *Z*-scores healthy control participants were within the normative range according to the published data from Bullard et al. (2004).

### T1 Structural Gray Matter Imaging Acquisition and Analysis

A structural T1-weighted 3-dimensional spoiled gradient-echo sequence was obtained on a Siemens 3.0T Trio scanner with an 8-channel head coil for 18 bvFTD and 13 CBS/PCA participants (sequence parameters: TR = 1620 ms, TE = 3 ms, flip angle = 15°, matrix = 192 × 256, slice thickness = 1 mm, and in-plane resolution = 1 × 1 mm). Reasons for exclusion included health and safety (e.g., metallic implants, shrapnel, claustrophobia), intercurrent medical illness, and lack of interest in an imaging study. Imaging was acquired on average within 125 (±75) days of performing the task. The subsets of patients for whom imaging data were available did not differ (*p* > 0.3) on any demographic, neuropsychological, or language performance measures from the total set of participants in each group. Imaging was also collected on 38 healthy controls. The three imaged groups were comparable in age and education, as determined by one-way ANOVAs [*F*(2,66) = 0.254, *p* = 0.777 and *F*(2,66) = 2.313, *p* = 0.107, respectively].

Gray matter images were normalized to a standard space and segmented using the PipeDream interface^1^ to the ANTS toolkit^2^ (Avants et al., 2014). The ANTS toolkit implements a diffeomorphic and symmetric registration and normalization method that is the most reliable tool available (Avants et al., 2008; Klein et al., 2010). A local T1 template with 1 mm isotropic resolution was built using ANTS from 25 healthy seniors and 25 frontotemporal lobar degeneration patients. Subject images were registered to the local template. The Atropos tool in ANTS (Avants et al., 2011) used template-based priors to guide three-tissue segmentation (GM, WM, and cerebrospinal fluid), and GM probability (GMP) images were calculated as a quantitative measure of GM atrophy. GMP images were then transformed into MNI space for statistical analysis and smoothed in SPM8^3^ using a 4-mm full-width half-maximum Gaussian kernel to minimize individual gyral variations. Finally, images were down-sampled to 2 mm isotropic resolution in order to attain a more anatomically relevant voxel size.

We used the *randomise* tool in FSL^4^ to perform two separate non-parametric, permutation-based statistical analysis (permutations = 10,000) to compare GM density between bvFTD patients and controls and CBS/PCA patients and controls. Analyses were restricted to voxels containing GM using the same explicit mask generated from the average GMP map of all imaged subjects. For each density comparison, we considered only clusters that exceeded an extent threshold of 50 voxels and a height threshold of *p* < 0.0001 (Bonferroni-corrected for multiple comparisons with threshold free cluster enhancement). We then used the *randomise* tool to perform regression analyses that related GM density to the BCET mean *Z*-score for each patient group (permutations = 10,000). Regression analyses were restricted to areas of GM disease for each patient group, as determined by the respective GM density analyses. Clusters with a height threshold of *p* < 0.05 (uncorrected for multiple comparisons) and an extent threshold of 10 voxels were considered significant.

### Diffusion Tensor Imaging White Matter Acquisition and Analysis

Diffusion tensor imaging was available for the same patients with bvFTD (*N* = 18) and controls (*N* = 38) with T1 imaging, and 12 of the 13 CBS/PCA patients with T1 imaging. A 30-directional DWI sequence was collected using single-shot, spin-echo, diffusion-weighted echo planar imaging (FOV = 240 mm; matrix size = 128 × 128; number of slices = 70; voxel size = 2 mm isotropic; TR = 8100 ms; TE = 83 ms; fat saturation). Thirty volumes with diffusion weighting (*b* = 1000 s/mm2) were collected along 30 non-collinear directions, and either one or five volumes without diffusion weighting (*b* = 0 s/mm2) were collected per subject. The PipeDream processing pipeline utilized ANTS (Avants et al., 2014) and Camino (Cook et al., 2006) to preprocess DWI. Motion and distortion artifacts were removed using affine co-registration of each diffusion-weighted image to the average of the unweighted (*b* = 0) images. Using a weighted linear least-squares algorithm (Salvador et al., 2005) implemented in Camino, DTs were computed. FA was computed from the DT image, and distortion between the subject’s T1 and DT image was corrected by registering the FA to the T1 image. DT images were then relocated to the local template (mentioned above) for statistical analysis by applying the FA-to-T1 and T1-to-local template warps, and tensors were reoriented using the preservation of principal direction algorithm (Alexander et al., 2001). Each participant’s FA image was recomputed from the DT image in local template space and smoothed using a 4 mm full-width half-maximum isotropic Gaussian kernel.

Healthy WM is vital for communication between different brain regions. Therefore, disruption in any WM tracts associated with the GM regions related to BCET performance may contribute to impaired cognition by disrupting the large-scale GM and WM network underlying cognitive estimation. Using group-level FA comparisons, we assessed the integrity of the WM tracts that integrate the GM regions associated with each patient group’s BCET mean *Z*-score regression analysis. To do this, we first inflated the GM regression results 3 voxels in all directions to assure that the regression clusters extended into the WM. We then performed two deterministic tractography procedures (maximum angle over 5 mm = 75°, minimum FA considered WM = 0.25) to identify all WM tracts associated with the GM regression results for each of the two patient groups (see Supplementary **Figure S1** for results of tracking procedure). The DT image used to generate the streamlines for both deterministic tractography images was a healthy senior population template image, created by averaging each DT image after its preprocessing and spatial normalization to the local template (as above). Next, we converted each tractography image into a volumetric WM image, one capturing the WM regions associated with the GM regression results of the patients with bvFTD, and one capturing the WM regions associated with the GM regression results for the patients with CBS/PCA. These volumetric WM images were used as masks for two FA comparisons that were performed using the *randomise* tool in FSL^5^ (permutations = 10,000), one between patients with bvFTD and controls, and one between patients with CBS/PCA and controls. For each FA comparison, we considered only clusters that exceeded an extent threshold of 50 voxels and a height threshold of *p* < 0.001 (uncorrected).

## Results

### Behavioral Results

To assess participant performance on the BCET, we calculated the mean *Z*-score across all questions. Two participants did not respond to all questions, therefore the mean *Z*-score for their responses did not include the questions that were not answered. The analyses were performed comparing each group separately because disease in specific neuroanatomical regions for each group was thought to lead to distinct forms of estimation impairment. Inspection of Q–Q Plots revealed that the distribution of scores was not normal. This was confirmed using a Shapiro–Wilks test of Normality. Therefore, non-parametric tests were used for all analyses.

**FIGURE 1:**
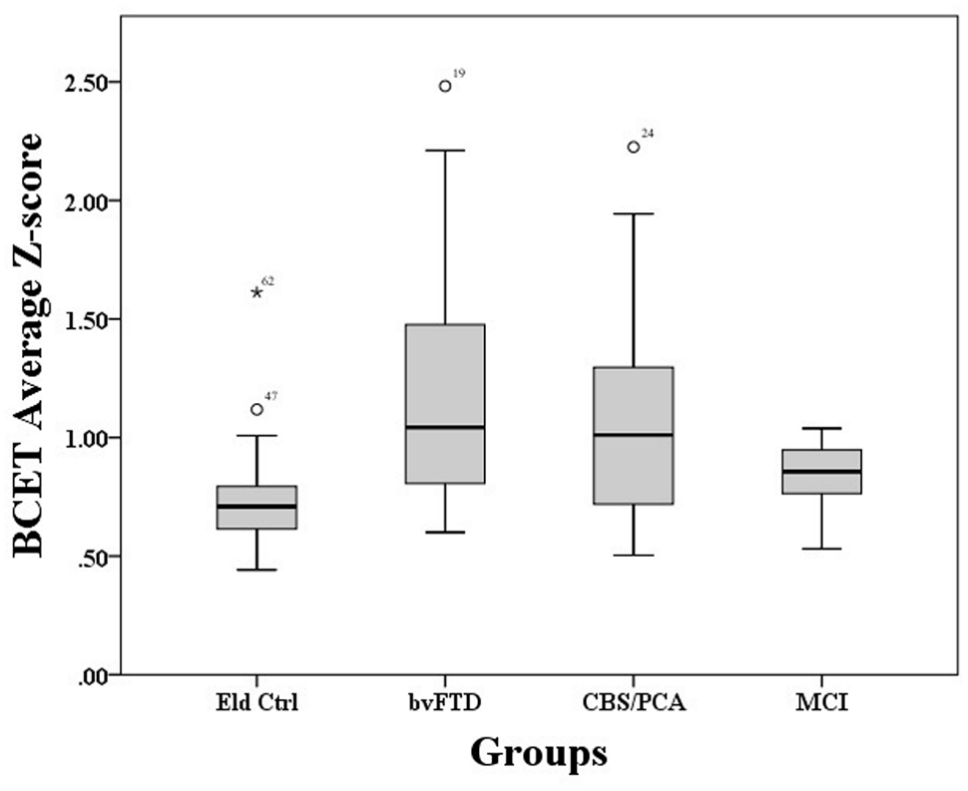
**Mean *z*-scores across all Biber cognitive estimation test questions for each group**.

A Kruskal–Wallis test showed that performance on the BCET differed between the four groups, χ^2^(3) = 15.22, *p* = 0.002. As illustrated in **Figure 1**, pairwise *post hoc* comparisons using Mann–Whitney with Bonferroni correction revealed that both the bvFTD [*U*(45) = 108.00, *p* < 0.001] and CBS/PCA [*U*(40) = 115.00, *p* = 0.012] groups differed from controls; however, the MCI group did not differ from controls, (*U*(34) = 88.00, *p* = 0.093). Further comparisons revealed that the bvFTD and CBS/PCA groups did not differ from each other in their performance on the BCET (*U*(37) = 164.00, *p* = 0.52). To confirm that the results of the CBS/PCA group were not driven by only one of those groups, we conducted a Mann–Whitney test comparing BCET performance in the PCA patients and the CBS patients and found no significant difference in performance between the two groups [*U*(15) = 29.00, *p = 0.69*].

To explore whether BCET impairment could be related to disease severity, correlations were conducted between BCET mean *Z*-scores and patient MMSE scores. These correlations were not significant for any of the patient groups (bvFTD: *r* = 0.11, *p* = 0.63; CBS/PCA: *r* = -0.40, *p* = 0.11; MCI: *r* = -0.42, *p* = 0.20).

### Structural Gray Matter Imaging Results

**FIGURE 2:**
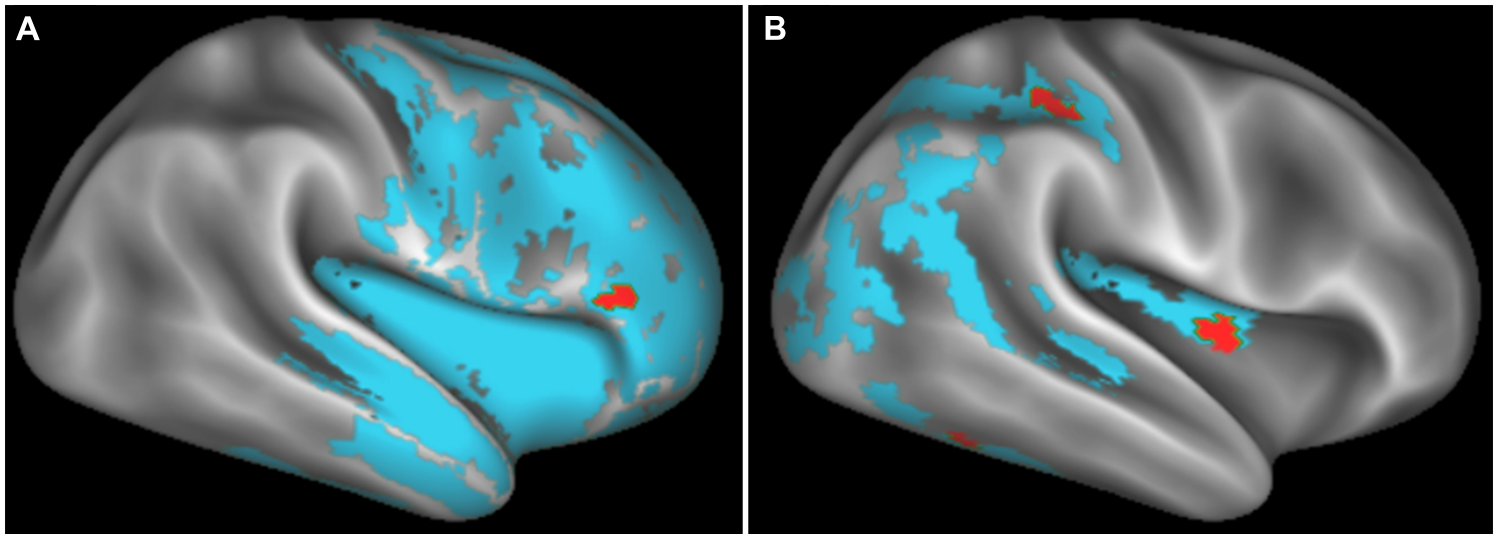
**Regions of significantly reduced GM density relative to controls (blue) and regression relating GM density to task performance (red) for behavioral variant frontotemporal degeneration **(A)** and corticobasal syndrome and posterior cortical atrophy (B)**.

Significant regions of reduced GMP and regression relating GMP to task performance for bvFTD and CBS/PCA patients are shown in **Figure 2**. Peak coordinates of GM atrophy and regression analyses are summarized in **Tables 2** and **3** respectively. MRI data for the MCI group was not analyzed as this group was not impaired on the BCET.

.

**Table 3:** Anatomic locations of significant GM atrophy.

Anatomic locus (Brodmann area)	MNI Coordinates			*P*-value	Cluster Size (voxels)
	*x*	*y*	*z*		
**bvFTD < Eld**
L insula	−34	22	6	<0.001	21104
R dorsolateral prefrontal (46)*	40	26	28	<0.001	n/a
R insula*	38	8	2	<0.001	n/a
R anterior cingulate (32)*	2	40	4	<0.001	n/a
L thalamus	−18	−32	0	<0.001	849
L middle temporal (21)	−52	4	−32	<0.001	74
**CBS/PCA < Eld**
R inferior parietal (40)	36	−48	42	<0.001	286
R middle occipital (18)	36	−82	8	<0.001	893
R fusiform (37)	48	−44	−26	<0.001	209
R insula	34	−20	16	<0.001	323

bvFTD, behavioral variant frontotemporal dementia; CBS, corticobasal syndrome; PCA, posterior cortical atrophy. *Subpeak at this location.

**Figure 2** illustrates significant GM atrophy in bvFTD patients that is apparent bilaterally in lateral frontal, medial frontal, anterior temporal, and medial temporal cortices. Coordinates for peak voxels are provided in **Table 3**, suggesting more extensive atrophy on the right. Peak coordinates are provided in **Table 4** for the regression analysis relating BCET mean *Z*-score cognitive estimation performance to reduced GMP. As illustrated in **Figure 2**, this implicated right lateral prefrontal cortex in cognitive estimation in bvFTD, as well as right orbital frontal, right hippocampus and left thalamus.

**Table 4:** Regressions of task performance with GM atrophy.

Anatomic locus (Brodmann area)	MNI Coordinates			*P*-value	Cluster Size (voxels)
	*x*	*y*	*z*		
**bvFTD mean *Z*-score**
R dorsolateral prefrontal (46)	52	32	10	0.026	11
R orbital frontal (11)	12	60	−10	0.002	14
R hippocampus	28	−38	0	0.024	23
L thalamus	−12	−28	0	0.008	11
**CBS/PCA mean *Z*-score**
R inferior parietal (40)	36	−30	38	0.013	18
R fusiform (37)	48	−48	−26	0.014	24
R insula	36	−6	8	0.005	11

*bvFTD, behavioral variant frontotemporal dementia; CBS, corticobasal syndrome; PCA, posterior cortical atrophy.*

In the CBS/PCA patient group, **Figure 2** shows that atrophy is restricted to the right hemisphere, focused in parietal, occipital, insular, and superior and posterior temporal cortices. Peak voxels for the atrophy analysis are summarized in **Table 3**. The regression analysis in CBS/PCA, summarized in **Figure 2** and **Table 4**, related reduced cognitive estimation performance to GMP in right inferior parietal cortex, as well as right insula and fusiform cortices.

### White Matter Imaging Results

**FIGURE 3:**
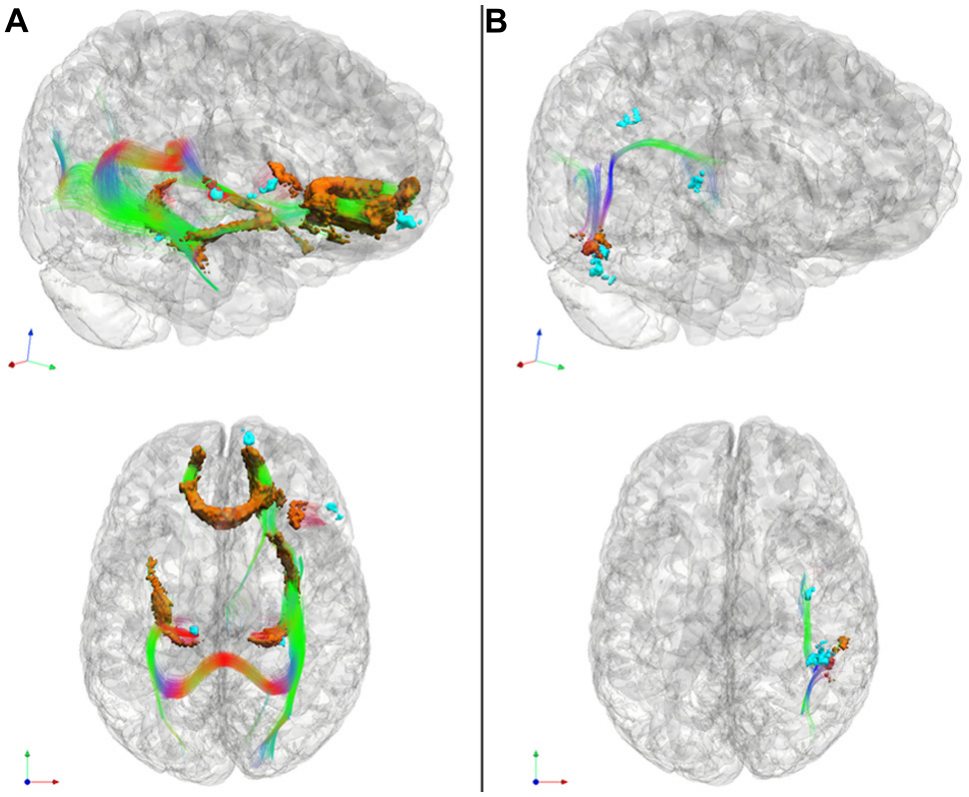
**Significantly reduced FA in WM tracts (RGB) associated with GM atrophy regions related to cognitive estimation task performance (cyan) for patients with behavioral variant frontotemporal degeneration **(A)** and for patients with corticobasal syndrome and posterior cortical atrophy (B)**. We also illustrate WM regions with reduced FA (orange). Please refer to **Table 5**. *Red: left–right, green: anterior–posterior, blue: inferior–superior.

After determining the GM regions related to cognitive estimation deficits in patients with bvFTD and patients with CBS/PCA, we examined the WM tracts related to GM atrophy to determine if they also displayed disease. As illustrated in **Figure 3**, we observed distinct patterns of reduced FA (shown in orange) relative to controls for each of the patient groups. This showed that WM associated with the GM regions related to cognitive estimation mean *Z*-score is also diseased. We display the tracts from the tractography analysis that are associated with (i.e., pass through) the reduced FA clusters (**Figure 3**, RGB streamlines). Peak coordinates of reduced FA are summarized in **Table 5**.

**Table 5:** Anatomic locations of reduced FA.

Anatomic locus	MNI Coordinates			*P*-value	Cluster Size (voxels)
	*x*	*y*	*z*		
**bvFTD < Eld**
Genu of corpus callosum	−13	30	0	<0.001	4373
R crus of fornix/stria terminalis	23	−36	8	<0.001	1552
L inferior longitudinal fasciculus	−38	−14	−16	<0.001	931
R inferior frontal gyrus WM	31	30	11	<0.001	206
**CBS/PCA < Eld**
R inferior temporal gyrus WM	52	−51	−12	<0.001	125
R superior longitudinal fasciculus	56	−40	−10	<0.001	113

*bvFTD, behavioral variant frontotemporal dementia; CBS, corticobasal syndrome; PCA, posterior cortical atrophy.*

In patients with bvFTD, there is significantly reduced FA in WM related to the regions implicated in the regression analysis relating GM atrophy to cognitive estimation performance. These regions include bilateral genu of the corpus callosum, the ILF, the rostral portion of the inferior fronto-occipital fasciculus, as well as WM in right inferior frontal gyrus that is proximal to the right inferior frontal cortical region implicated in the GM regression analysis with cognitive estimation performance.

Patients with CBS/PCA also show reduced FA in WM regions related to results of regression analysis of GM atrophy and cognitive estimation performance. However, in contrast to the patients with bvFTD, the patients with CBS/PCA displayed regions of significantly reduced FA in WM of the right temporal-parietal region, including vertical portions of the SLF.

We did not observe a significant correlation between FA and BCET performance.

## Discussion

Cognitive estimation is a complex process that we use commonly in the face of imprecise knowledge about quantities associated with familiar objects. Central components of cognitive estimation include executive resources needed to formulate a reasonable probability, and knowledge of numbers. We found that patients with bvFTD and CBS/PCA, but not MCI, are significantly impaired with cognitive estimation. Thus, they have difficulty estimating the number of slices in a loaf of bread, and other common, day-to-day estimates involving quantity, time and distance. This appears to be due in part to disease that compromises a frontal-parietal network that plays a crucial role in probabilistic and quantitative components of cognitive estimation. We consider each of these issues below.

Patients with bvFTD have significant deficits with executive resources and social functioning. This results in one of the cardinal clinical features of bvFTD – an impairment of judgment that manifests clinically as inappropriate behaviors largely unmodulated by social norms (Rascovsky et al., 2011). Difficulty estimating the range of appropriate responses in a social situation is analogous to the difficulty that we observe when bvFTD patients are attempting to estimate a quantity associated with a familiar object like a loaf of bread. A range of responses is provided by healthy controls when asked to estimate a quantity such as the number of slices in a loaf of bread. This is because we generally do not have a precise representation of quantities associated with these kinds of objects. Nevertheless, we can provide a reasonable estimate that is close to the actual quantity. This can be inferred semi-quantitatively through a multistep process such as estimating the thickness of a bread slice, estimating the length of a loaf of bread, and then estimating the number slices in the loaf. Alternately, a reasonable approximation can be offered that is within the rough Poisson distribution of one’s experiences with a loaf of bread. Regardless of the specific basis for estimation, patients with bvFTD tend to provide an estimate that is more distant from the average quantity provided by a reference population of young healthy controls. For example, evidence has been shown suggesting that bvFTD patients have difficulty developing a multistep strategy that can support the estimation process (Carey et al., 2008; Johns et al., 2009; Poletti et al., 2011), and it is possible that this strategic deficit limits the ability to formulate a reasonable estimation. Alternately, fMRI work has shown that probability estimation activates dlPFC (Casey et al., 2001), an area that is compromised in bvFTD, and these patients may have difficulty with developing a Poisson distribution that captures the likelihood of a particular quantity associated with the target concept. While some work has shown a deficit in number knowledge in bvFTD patients (Halpern et al., 2003), it is less likely that this can explain the estimation impairment. The number deficit in these patients thus appeared to depend specifically on the performance of complex calculations rather than a deficit in number knowledge. It is unlikely that these patients did not understand the target concept since there was no correlation between their estimation performance and measures on tasks requiring semantic memory. While bvFTD patients can be apathetic or distractible, these characteristics are unlikely to account for their poor estimates of quantity since their responses were not totally unrelated to the range of quantities for the estimation; for example, they never responded with a very small number like “1” or a three-digit quantity when asked to estimate the number of slices in a bread loaf. It does not appear that working memory limitations can explain their deficit because they were asked to judge only one object concept at a time and there was no interference that could have compromised their ability to hold the concept in mind while they were making a judgment.

Patients with bvFTD have disease predominantly in prefrontal regions of the brain. Many studies have associated deficits on measures of executive functioning with atrophy of the frontal lobe (Manes et al., 2002; Kramer et al., 2003; Alvarez and Emory, 2006; Libon et al., 2009). In the present study, we found that frontal lobe atrophy is associated with impaired cognitive estimation as well. In particular, regression analyses associated their deficit in cognitive estimation with atrophy in dlPFC and orbital frontal cortex. dlPFC is an area that has been associated with probability estimation in fMRI studies of healthy controls (Casey et al., 2001). This area also has been associated with the strategic development of multistep planning such as that required to perform a task like the Tower of London (Baker et al., 1996; Newman et al., 2003). Orbital frontal cortex has been associated with mental flexibility (Milner, 1963; Kim and Ragozzino, 2005; Eslinger et al., 2007; Stalnaker et al., 2007; Evans et al., 2015), and disease in this area may be contributing to performance by limiting the ability to assess more than a limited range of possible quantitative estimates.

There is also disease in WM regions in bvFTD, and this appears to contribute to the cognitive estimation deficit in these patients. WM disease in the anterior corpus callosum may interrupt information transfer between hemispheres. Disease in ILF may interfere with relating visual or imaged representations of objects in visual association cortex to frontal regions important for estimation. Diseased U-fibers in orbital frontal regions may be important for mental flexibility.

Cognitive estimation also depends in part on number knowledge. The current study shows that patients with CBS/PCA have difficulty with cognitive estimation; we attribute their deficit to limited knowledge of numbers. The estimation process thus is being applied over the domain of a quantitative property of a familiar object, and knowledge of quantity is necessary to support this process. Much work has demonstrated a significant deficit in number knowledge in these patients (Halpern et al., 2004; Koss et al., 2010; Morgan et al., 2011; Spotorno et al., 2014). Some have observed executive limitations in patients with CBS (Huey et al., 2009), and we cannot rule out that this also may be contributing to their deficit.

Many imaging studies (Whitwell et al., 2007, 2010b; Lehmann et al., 2011) and autopsy-verified assessments (Alladi et al., 2007; Murray et al., 2007; Pantelyat et al., 2011) underline the distribution of disease in the parietal lobe in CBS/PCA. Extensive fMRI work in healthy adults has associated the parietal lobe with number knowledge (Rueckert et al., 1996; Pinel et al., 1999), and thus it is not surprising that these patients have difficulty with processing a quantity – in this case, developing a quantity estimation. Consistent with these findings, the regression analysis found that cognitive estimation in CBS/PCA is related to cortical atrophy in the right inferior parietal lobule. The insula may be playing a role in evaluating the value of a correct response on this measure (Singer et al., 2009). We also observed WM disease in CBS/PCA that is associated with these GM regions. Disease in the vertical portion of the SLF and posterior temporal regions may contribute to relating visual or imaged object representations processing in visual association cortex to parietal regions important for quantity processing.

Specificity for our proposed fronto-parietal network comes from our observation that MCI patients, our dementia control group, were not impaired in their estimation abilities. This observation suggests that cognitive estimation difficulties are related to a specific neuroanatomic network and that our results are not simply related to non-specific or global neurodegeneration of the brain. These participants were matched with the focal patient groups for MMSE, a brief neuropsychological evaluation of cognitive functioning. Furthermore, we did not find a significant correlation relating disease severity to estimation abilities. These results further support our hypothesis that a network specifically involving frontal and parietal regions is involved in cognitive estimation.

Several caveats should be kept in mind when evaluating our findings. We examined a small number of patients, and a larger cohort is needed to evaluate estimation performance. Because of power limitations, we used a relatively liberal threshold to relate performance to regional cortical atrophy. Nevertheless, we believe that these findings are likely to be valid because regressions are in areas of known disease for each patient group, the regressions encompass areas of disease consistent with predictions based on previous work, and the findings are unique to each group. We also did not observe a direct correlation between FA and BCET performance. It is possible that heterogeneity between groups such as the amount of WM disease in individuals associated with distinct underlying pathological sources (McMillan et al., 2013) may have obscured our findings. Additional work in larger and pathologically confirmed samples are necessary to further explore the potential contributions of WM to cognitive estimation. While regression analyses relating performance to structural imaging provide converging evidence implicating the estimation process and knowledge of quantity in the estimation deficit in these patients, it would be useful to have additional evidence about estimation with this task from other sources such as fMRI activation or rTMS in healthy adults. With these caveats in mind, our findings are consistent with the hypothesis that a frontal-parietal network plays a central role in the process of cognitive estimation. Based on studies of patients with focal neurodegenerative disease, we observed that patients with predominantly prefrontal disease and patients with predominantly parietal disease have difficulty with estimating a quantitative property of a familiar object. This is consistent with the claim that a large-scale frontal-parietal network plays a crucial role in cognitive estimation.

## Author Contributions

All authors participated in drafting and revising the manuscript for intellectual content.

## Conflict of Interest Statement

The authors declare that the research was conducted in the absence of any commercial or financial relationships that could be construed as a potential conflict of interest.

## Footnotes

1 http://www.sourceforge.net/projects/neuropipedream/

2 http://www.picsl.upenn.edu/ANTS/

3 http://www.fil.ion.ucl.ac.uk/spm/software/spm8

4 http://fsl.fmrib.ox.ac.uk/fsl/fslwiki/Randomise

5 http://fsl.fmrib.ox.ac.uk/fsl/fslwiki/Randomise

## Supplementary material

Figure S1 **Tracts used in RGB mask for comparisons of fractional anisotropy for behavioral variant frontotemporal degeneration (A) and for the combined corticobasal syndrome and posterior cortical atrophy (B).** Red: left–right, green: anterior–posterior, blue: inferior–superior

## Abbreviations

BCET: Biber cognitive estimation test
BNT: Boston naming test
bvFTD: behavioral variant frontotemporal dementia
CBS: corticobasal syndrome
dlPFC: dorsolateral prefrontal cortex
DT: diffusion tensor
DTI: diffusion tensor imaging
DWI: diffusion weighted imaging
FA: fractional anisotropy
GM: gray matter
GMP: gray matter probability
MMSE: mini-mental state examination
PBAC: Philadelphia Brief Assessment of Cognition
PCA: posterior cortical atrophy
PFC: prefrontal cortex
PPT: pyramid and palm tree test
WM: white matter.
