# Supplementary figures and images for: Estimating frontal and parietal involvement in cognitive estimation: a study of focal neurodegenerative diseases
Source: Front Hum Neurosci. 2015 Jun 4;9:317. doi: 10.3389/fnhum.2015.00317 (PMC4454843; doi:10.3389/fnhum.2015.00317)

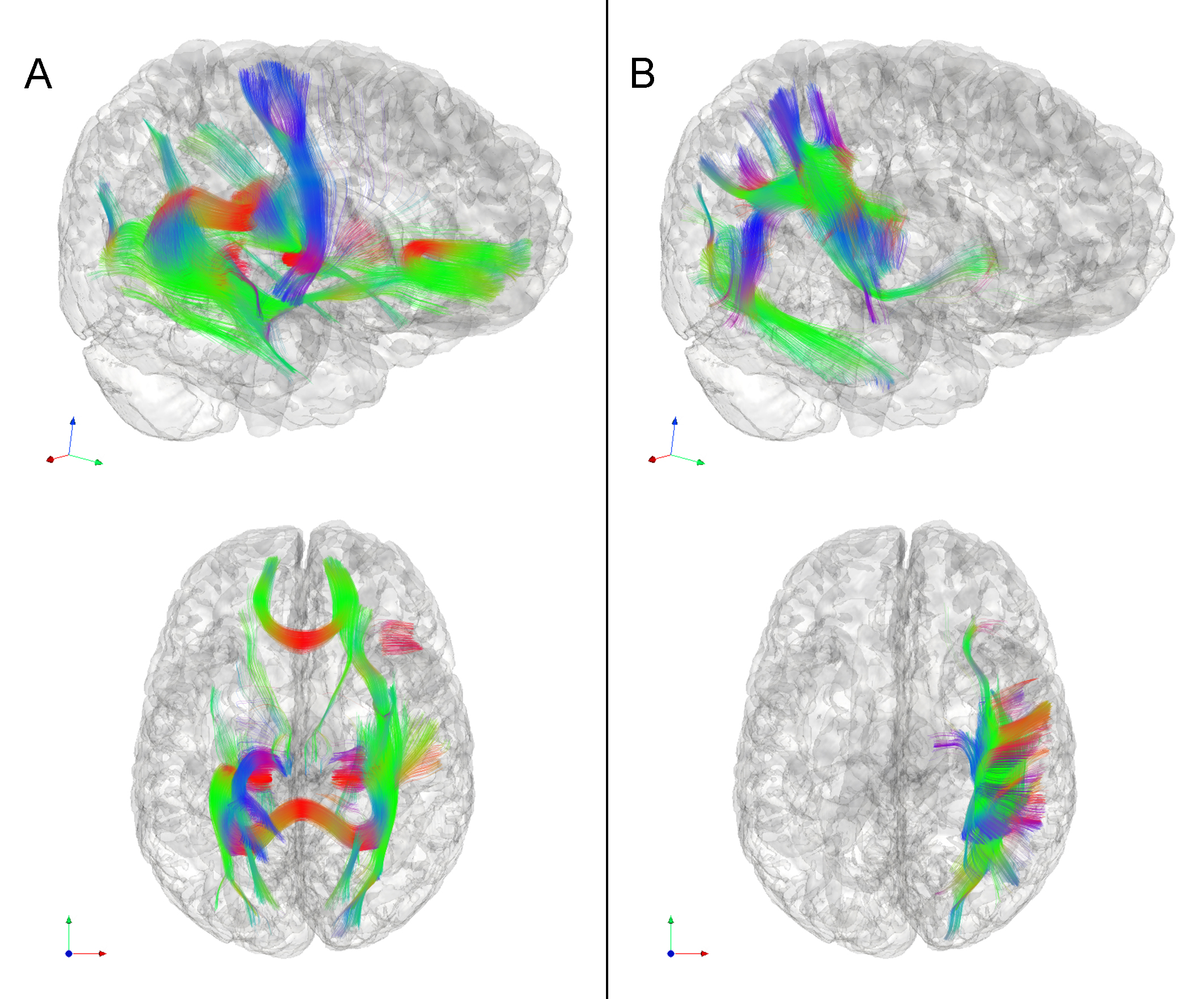

Supplement: Figure S1 — Tracts used in RGB mask for comparisons of fractional anisotropy for behavioral variant frontotemporal degeneration (A) and for the combined corticobasal syndrome and posterior cortical atrophy (B). Red: left–right, green: anterior–posterior, blue: inferior–superior [file Image_1.JPEG]
